# RT-QuIC Using C-Terminally Truncated α-Synuclein Forms Detects Differences in Seeding Propensity of Different Brain Regions from Synucleinopathies

**DOI:** 10.3390/biom11060820

**Published:** 2021-05-31

**Authors:** Ilaria Poggiolini, Daniel Erskine, Nishant N. Vaikath, Janarthanan Ponraj, Said Mansour, Christopher M. Morris, Omar M. A. El-Agnaf

**Affiliations:** 1 Neurological Disorder Research Center, Qatar Biomedical Research Institute (QBRI), Hamad Bin Khalifa University (HBKU), Qatar Foundation, Doha 974, Qatar; ilaria.poggiolini@gmail.com (I.P.); nvaikath@hbku.edu.qa (N.N.V.); 2 Translational and Clinical Research Institute, Newcastle University, Newcastle upon Tyne NE2 4AA, UK; Daniel.Erskine@newcastle.ac.uk; 3 Core Labs, Qatar Environment and Energy Research Institute (QEERI), Hamad Bin Khalifa University (HBKU), Qatar Foundation, Doha 974, Qatar; jponraj@hbku.edu.qa (J.P.); smansour@hbku.edu.qa (S.M.); 4 Newcastle Brain Tissue Resource, Translational and Clinical Research Institute, Campus for Ageing and Vitality, Newcastle University, Newcastle upon Tyne NE4 5PL, UK; c.m.morris@newcastle.ac.uk

**Keywords:** synucleinopathies, Parkinson’s disease, dementia with Lewy bodies, RT-QuIC, α-synuclein, C-terminally truncated αSyn

## Abstract

Aggregated α-synuclein (αSyn) protein is a core pathological feature of Parkinson’s disease (PD) and dementia with Lewy bodies (DLB). Both PD and DLB demonstrate the presence of diverse intracellular α-synuclein (αSyn) species, including C-terminally truncated αSyn (C-αSyn), although it is unknown how C-αSyn species contribute to disease progression. Using recombinant C-αSyn and PD and DLB brain lysates as seeds in the real-time quaking-induced conversion (RT-QuIC) assay, we explored how C-αSyn may be involved in disease stratification. Comparing the seeding activity of aqueous-soluble fractions to detergent-soluble fractions, and using αSyn 1-130 as substrate for the RT-QuIC assay, the temporal cortex seeds differentiated PD and DLB from healthy controls. In contrast to the temporal cortex, where PD and DLB could not be distinguished, αSyn 1-130 seeded by the detergent-soluble fractions from the PD frontal cortex demonstrated greater seeding efficiency compared to the DLB frontal cortex. Moreover, proteinase K-resistant (PK^res^) fragments from the RT-QuIC end products using C-αSyn 1-130 or C-αSyn 1-115 were more obvious in the frontal cortex compared to the temporal cortex. Morphological examinations of RT-QuIC end products showed differences in the size of the fibrils between C-αSyn 1-130 and C-αSyn 1-115, in agreement with the RT-QuIC results. These data show that C-αSyn species can distinguish PD from DLB and suggest diversity in αSyn species across these synucleinopathies, which could play a role in disease progression.

## 1. Introduction

Parkinson’s disease (PD), dementia with Lewy bodies (DLB), and multiple system atrophy (MSA) [1] represent the primary synucleinopathies, an umbrella term for a group of disorders related to the aggregation of α-synuclein protein (αSyn). The misfolding and aggregation of αSyn in neurons, neuronal processes, or glial cells is thought to be a critical pathogenic event in synucleinopathies. In PD and DLB, αSyn inclusions are detected as Lewy bodies (LBs) and Lewy neurites (LNs) in subcortical and cortical neurons, while in MSA, αSyn inclusions mainly manifest in glial cells and are referred to as glial cytoplasmic inclusions (GCIs) [2,3]. The presence of αSyn aggregates in specific cell types is thought to underlie the phenotypic differences between MSA and the other synucleinopathies. These differences in presentation are potentially due to variable solubilities of αSyn species in specific synucleinopathies and have been proposed to account for the existence of different αSyn strains able to spread from cell to cell in a prion-like manner [4,5,6]. Moreover, different synucleinopathies show regional variation in the brain of both the initiation and progression of αSyn pathology, which might in turn affect the intrinsic structure of αSyn aggregates [7,8].

Recently, in vitro αSyn amplification techniques, including the protein misfolding cyclic amplification (PMCA) assay and real-time quaking-induced conversion (RT-QuIC) assay, have been used to discriminate between synucleinopathies [7,9,10,11]. In these assays, pathogenic αSyn-containing samples (αSyn ‘seeds’) are added to reaction mixtures containing recombinant full-length αSyn monomers. These assays take advantage of the capacity of aggregated αSyn to induce the misfolding and incorporation of αSyn monomers, and the ability of fibrillar conversion products to be recognized by the amyloid fibril-sensitive fluorescent dye, thioflavin T (ThT). Through seeded conversion, monomeric recombinant full-length αSyn grows into amyloid fibrils that specifically bind to ThT and thus αSyn conversion can be monitored in real-time by measuring ThT fluorescence. Recent studies have reported controversial results about the ability of aggregation assays to stratify between different synucleinopathies. While Rossi et al. [12] reported no differences in the seeding assay response between LBs from related syndromes with no αSyn seeding activity in MSA cerebrospinal fluid (CSF) samples, Shahnawaz et al. [10] showed that patients with different synucleinopathies can be distinguished in PMCA on the basis of the αSyn strain seeding propensity with MSA CSF and brain samples aggregating faster but reaching a lower fluorescence plateau than PD CSF and brain samples. The comparison of PMCA-derived strains has been shown to mirror the differences in synucleinopathy phenotype, with PD and MSA brain PMCA-derived fibrils sharing similar biochemical features that are distinct from those of DLB patient-derived fibrils [7]. Moreover, in cell culture distinct PMCA-amplified αSyn strains have been shown to seed monomeric αSyn aggregation to a different extent, and such differences may underlie the broad clinical spectrum of synucleinopathy phenotypes [7].

Some αSyn species present in human brain tissue are N- or C-terminally truncated, and these modifications have been demonstrated to enhance αSyn aggregation and neurodegeneration both in vitro and in vivo [13,14,15,16]. Around 15% of the total aggregated αSyn present in LBs is C-terminally truncated (C-αSyn) at positions proximal to residue 120 [11,13,16,17]. In vitro, physiologically C-terminally truncated αSyn forms increase both the extent and rate of αSyn fibril formation, and enhance the aggregation of full-length αSyn and mixed C-terminally truncated or full-length αSyn fibrils to sequester further full-length αSyn into aggregation [18]. It has been shown that the C-terminal truncation of αSyn protein can influence its prion-like pathogenicity and lead to the formation of different αSyn strains that may be responsible for the phenotypic diversity observed in different synucleinopathies [19]. Therefore, we hypothesized that C-terminal truncated αSyn, a putatively more aggregation-prone and disease-relevant species of αSyn, may better distinguish regional variabilities of αSyn seeding capacity across the brain and, thus, distinguish PD from DLB.

In this study, we sought to determine whether αSyn C-terminal truncations behave differently in an RT-QuIC-based assay and allow distinction between the different synucleinopathies in brain lysates derived from temporal and frontal cortices of DLB and PD patients. We compared the kinetics of aggregation of two C-terminal truncated αSyn species (αSyn 1-130 and αSyn 1-115) to that of full-length αSyn (αSyn 1-140). We also analyzed the RT-QuIC end products by proteinase K digestion and electron microscopy in order to gain further insights on the structures of the aggregates that are involved in different synucleinopathies.

## 2. Materials and Methods

### 2.1. Expression and Purification of Recombinant αSyn Proteins

Recombinant human full-length αSyn (1-140) (Fl-αSyn140) was expressed from the pRKl72 plasmid containing full-length cDNA for the human *SNCA* gene. Truncated recombinant αSyn 1-130 (C-αSyn130) and 1-115 (C-αSyn115) were expressed from pET3A plasmid containing the cDNA sequence as previously described [20]. Briefly, all the recombinant αSyn forms were expressed in E. Coli BL21 (DE3) and purified using size exclusion and Mono Q anion exchange chromatography. For αSyn 1-115, the Mono Q anion exchange elution buffer was prepared at pH 9 to account for an increasing isoelectric point in this truncated protein. All recombinant proteins were diluted in 20 mM Tris/HCl pH 7.4, 100 mM NaCl, and protein concentrations were determined using the bicinchoninic acid assay (Pierce). Aliquots (300 μL of 1 mg/mL) were prepared and stored at −80 °C. Prior to use, the proteins were filtered (100 kDa spin filter) and the protein concentration was again determined by bicinchoninic acid assay.

### 2.2. Circular Dichroism Spectra

Circular dichroism (CD) spectra were measured with a Chirascan CD Spectrophotometer (Applied Photophysics) using a quartz cell with a 1 mm path length. All measurements were carried out using 5 μM αSyn in PBS pH 7.4. The CD spectra were obtained averaging five scans in the wavelength range of 195–250 nm.

### 2.3. Isolation of TBS (aqueous)-Soluble and Detergent-Soluble Fractions from Brain Tissue

Brain tissues from PD or DLB cases, and healthy controls, were obtained from Newcastle Brain Tissue Resource, Newcastle University, UK (refer to Supplementary Table for clinical data). Extracts from the temporal or frontal cortex were prepared as previously described [20]. Briefly, samples were homogenized with a glass tissue homogenizer at 10% (*w*/*v*) on ice in TBS (20 mM Tris-HCl pH 7.4, 150 mM NaCl) and 5 mM EDTA with protease and phosphatase inhibitors (Thermo Fisher Scientific). Samples were centrifuged at 3000× *g*, at 4 °C for 30 min. The collected supernatant represents the TBS (aqueous)-soluble fraction. The pellet was then resuspended in CelLytic buffer (Sigma), homogenized as before on ice, and centrifuged at 3000× *g* at 4 °C for 30 min. The resulting supernatant represented the detergent-soluble fraction. The total protein concentration was measured in both fractions by BCA assay (Pierce, Thermo Fisher Scientific) and 0.1 mg/mL aliquots were prepared and stored at −80 °C.

### 2.4. RT-QuIC Assay

The RT-QuIC assay was performed using purified recombinant αSyn and re-optimized from the previously described method [21]. The reaction buffer was composed of 100 mM piperazine-N,N’-bis(ethanesulfonic acid) (PIPES; pH 6.9), 0.1 mg/mL αSyn, and 10 µM ThT. Reactions were performed in triplicate in black 96-well plates with a clear bottom (Nunc, Thermo Fischer) with 85 µL of the reaction mix loaded into each well together with 15 µL of 0.1 mg/ml TBS-soluble or detergent-soluble fractions. The plate was then sealed with a sealing film (Thermo Fisher Scientific) and incubated in a BMG LABTECH FLUOstar OMEGA plate reader at 37 °C for 100 h with intermittent cycles of 1 min shaking (500 rpm, double orbital) and 15 min rest throughout the indicated incubation time. ThT fluorescence measurements, expressed as arbitrary relative fluorescence units (RFU), were taken with bottom reads every 15 min using 450 ± 10 nm (excitation) and 480 ± 10 nm (emission) wavelengths. The final fluorescence value was the mean fluorescence value taken at 100 hours. A positive signal was defined as RFU more than 5 standard deviation units (RFU > 5 SD) above the mean of initial fluorescence. The sample was considered positive if at least two of the replicates were positive, otherwise the sample was classified as negative. The lag time was calculated as the time required to reach a positive signal (RFU > 5 SD); if a positive signal was not recorded then the lag time was set to 100 h. As a negative control, unseeded RT-QuIC reactions were prepared with recombinant αSyn only (i.e., without brain homogenates), which showed no aggregation (Supplementary Figure S1).

### 2.5. Proteinase K Digestion and Western Blotting

Proteinase K (PK) digestion of the reaction end products was performed using two different proteinase K concentrations (0.4 and 4 µg/mL) at 37 °C for 30 min. The reactions were stopped by adding NuPAGE LDS buffer and incubating the samples at 60 °C for 10 min. Samples were analyzed by electrophoresis in 4%–12% Bis-Tris gels (Invitrogen) using MES as running buffer and immunoblotted on nitrocellulose membranes (Amersham). Blots were blocked in PBS containing 0.05% (*v*/*v*) Tween 20 (PBST) and 5% (*w*/*v*) non-fat dried skimmed milk powder and probed with Syn1 antibody (aa91-99 of human αSyn) at 50 ng/mL final antibody concentration in PBST. Blots were developed using ECL detection Western blotting reagents (Pierce).

### 2.6. Transmission Electron Microscopy (TEM)

Samples of αSyn RT-QuIC end products were transferred in a 5 μL volume to a TEM grid (S162 Formvar/Carbon, 200 Mesh, agar scientific). After 1 min, samples were fixed using 5 μL of 0.5% glutaraldehyde followed by one wash with 50 µL of ddH_2_O. Five microliters of 2% uranyl acetate were added to the grid, and after two minutes uranyl acetate was blotted off and grids were dried before placing sample holders before visualization. Three TEM images (corresponding to the three PD or DLB cases) were taken for each condition tested (αSyn substrate, brain region, and extract type) and used for ImageJ quantification of the area of the fibril aggregates and the average length of 5 to 10 fibrils within each aggregate.

### 2.7. Statistical Analysis

All statistical analyses were performed using GraphPad Prism Software (Version 9.0.2, GraphPad Software, Inc., San Diego, CA, USA). The types of tests performed are detailed in the figure legends. *p*-values < 0.05 were considered statistically significant.

## 3. Results

### 3.1. Characterization of αSyn Forms Used as Substrates and Effect of Buffer Composition on the RT-QuIC Assay

**Figure 1:**
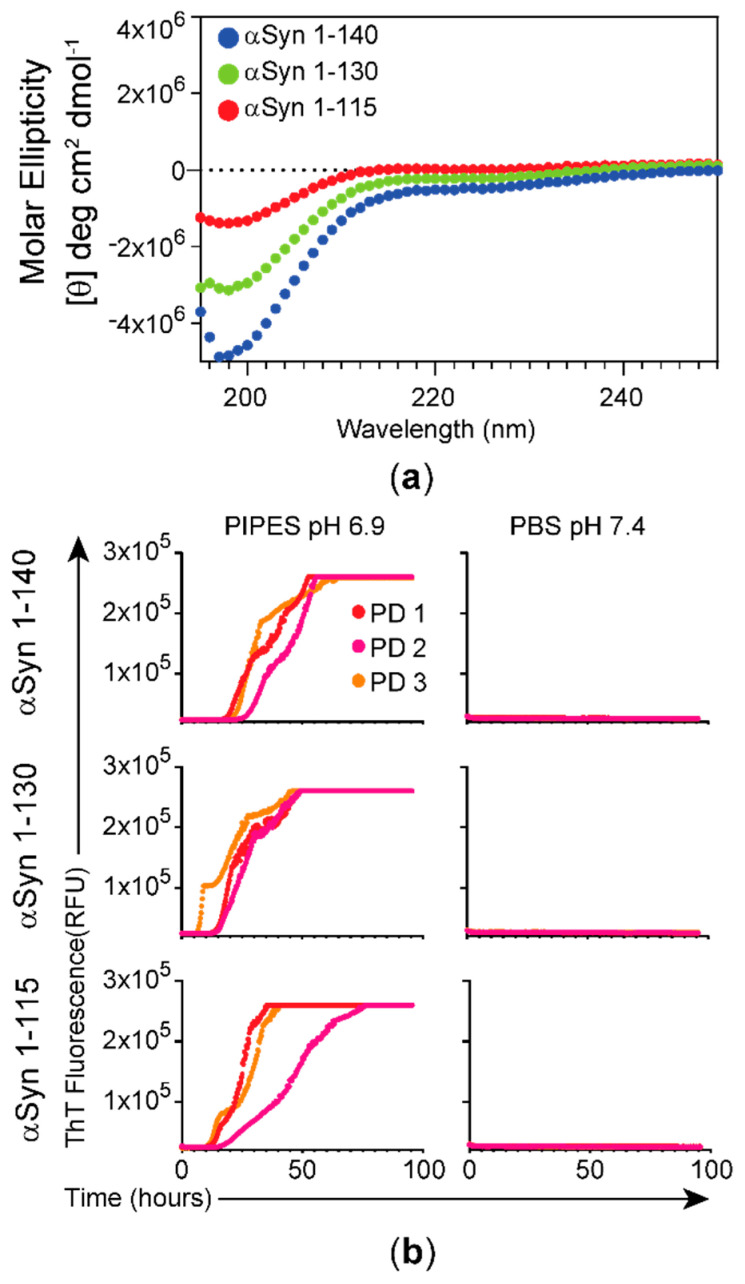
Characterization of αSyn monomeric substrates and RT-QuIC assay reaction buffer. (**a**) The secondary structure of the three monomeric recombinant αSyn forms tested by circular dichroism (CD) show a minimum mean residue ellipticity at 195 nm, typical of disordered proteins with random coil. (**b**) Effect of RT-QuIC reaction buffer on αSyn seeding activity. The RT-QuIC assay was performed in two different reaction mixtures (PIPES or PBS) for αSyn 1-140, αSyn 1-130, or αSyn 1-115 as substrates and the TBS fractions extracted from the temporal cortex samples from PD patients (n = 3) as seeds. The curve from each PD patient is the average of 3 technical replicates.

As the RT-QuIC assay is dependent on the quality of the substrate used in the reaction, we analyzed the recombinant αSyn monomers (αSyn) used in this study for their secondary structure by circular dichroism (CD). The CD spectra of the αSyn monomers showed negative bands at 195 nm, consistent with a random coil structure (Figure 1a), and the molar ellipticity increased with increasing truncation of the C-terminus of the recombinant protein; Fl-αSyn140 versus C-αSyn130 versus C-αSyn115. To optimize and implement the conditions for αSyn RT-QuIC, we tested two different reaction buffers, 100 mM piperazine-N,N’-bis(ethanesulfonic acid) (PIPES; pH 6.9) and 1X PBS (10 mM sodium phosphate, 138 mM NaCl, and 2.7 mM KCl; pH 7.4). As shown in Figure 1b, only 100 mM PIPES (pH 6.9) showed an increase in the ThT fluorescence curves in the RT-QuIC assays with three different cases of PD, which began to increase at 15, 24, and 40 post-reaction with C-αSyn115, C-αSyn130, and Fl-αSyn140, respectively.

### 3.2. Seeding RT-QuIC Reactions with Brain Homogenates from PD and DLB Cases Using Different Forms of αSyn

**Figure 2:**
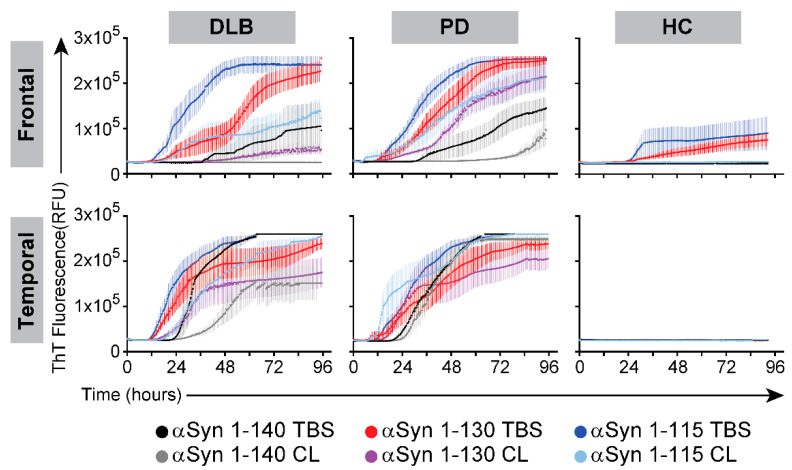
RT-QuIC assays with recombinant αSyn forms in RT-QuIC using brain homogenates from PD and DLB cases. Aqueous-soluble and detergent-soluble (CelLytic, CL) fractions were prepared from the temporal and frontal cortices of 8 cases of DLB, PD, or healthy controls (HC), then assayed by RT-QuIC using three αSyn forms (full length 1-140 and truncated forms 1-130 or 1-115) as described in Section 2. Data shown are the averages (±SEM) of ThT signal (relative fluorescence units, RFU) over time.

PIPES pH 6.9 was selected as the optimal working buffer and used to compare the three forms of αSyn in RT-QuIC assays. We sequentially prepared extracts from the brain regions with TBS followed by non-ionic detergent (CelLytic) to take into account the variable degree of solubility of the different amyloidogenic species present in brain samples [20,22]. The average curves from the RT-QuIC assays are shown in Figure 2 for reactions carried out with TBS-soluble or detergent-soluble fractions from the temporal or frontal cortices from PD or DLB cases against healthy controls (HC).

There was a trend for faster reaction kinetics with increasing C-terminal truncation of αSyn, particularly for reactions seeded with extracts from the frontal cortex samples where the longest lag-phases were observed with Fl-αSyn140 with aqueous-soluble fractions (45 ± 21 h for PD, 59 ± 18 h for DLB, *p* > 0.05) and detergent-soluble fractions (54 ± 6.4 h for PD). The shortest lag-phases in the frontal cortex were seen with C-αSyn115 with aqueous-soluble fractions (20 ± 5.4 h for PD, 20 ± 6 h for DLB) and detergent-soluble fractions (38 ± 30 h for PD, 54.5 ± 29 h for DLB). The lag-phase was significantly shorter with C-αSyn115 than Fl-αSyn140 in the reactions seeded with the aqueous-soluble and detergent-soluble fractions extracted from the frontal cortex samples (*p* < 0.05 and *p* < 0.01, respectively).

For a quantitative assessment, we defined the area under the RT-QuIC curves (AUC) as the seeding parameter of interest as it summarizes all the kinetic features of the aggregation reaction, including the speed and extent of aggregation. The RT-QuIC AUC from the frontal cortices did not correlate with temporal cortex samples from the same case in any of the tested fractions/substrate combinations (data not shown), supporting that the differences in the AUC are specific to the different brain regions.

**Figure 3:**
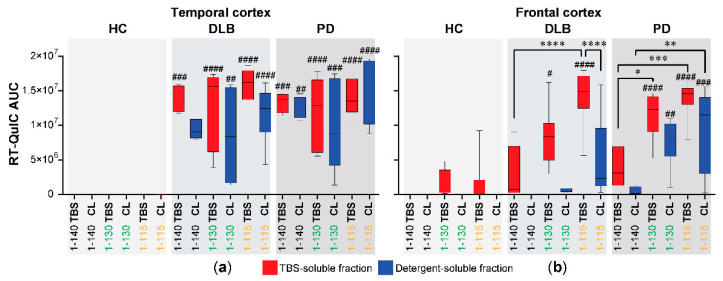
Comparisons of the aggregation of αSyn forms in RT-QuIC using brain homogenates from PD and DLB cases. TBS-aqueous and detergent-soluble (CelLytic, CL) fractions were prepared from (**a**) temporal and (**b**) frontal cortices of 8 cases of DLB, PD, or healthy controls (HC), then assayed by RT-QuIC using three different αSyn forms (full length 1-140 and truncated forms 1-130 or 1-115). The RT-QuIC reactions are shown in Figure 2. The box plots (median marked with a line and with maximum and minimum bars) summarize the area under the RT-QuIC curves (AUC). One-way ANOVA with Tukey’s multiple comparison testing (GraphPad Prism) was used for statistical comparisons. Hashtags denote significant differences in comparison to healthy controls (HC); ^#^ *p* < 0.05, ^##^ *p* < 0.01, ^###^ *p* < 0.001, ^####^ *p* < 0.0001. Asterisks denote significant differences within DLB or PD; * *p* < 0.05, ** *p* < 0.01, *** *p* < 0.001, **** *p* < 0.0001.

The temporal cortex from DLB and PD showed significantly higher AUC for the three forms of αSyn used compared to healthy controls (*p* < 0.05) and without significant differences between the αSyn forms or between the TBS-soluble and detergent-soluble fractions (Figure 3a). On the other hand, the frontal cortex from DLB and PD showed more variations (Figure 3b). In the TBS-soluble fractions from frontal cortices, aggregation with C-αSyn115 was significantly faster than Fl-αSyn140 in DLB, while both C-αSyn130 and C-αSyn115 had a significantly faster aggregation rate than Fl-αSyn140 in the PD (marked by asterisks in Figure 3b). In the detergent-soluble fractions from the frontal cortices, only C-αSyn115 with PD had significantly faster aggregation compared to Fl-αSyn140 (** *p* < 0.01). Finally, the only significant difference between the TBS- and detergent-soluble fractions from the frontal cortex was observed with C-αSyn115 in DLB (Figure 3b, *p* < 0.0001).

Collectively, the RT-QuIC data suggested that in the temporal cortex (Figure 3a), both TBS- and detergent-soluble fractions from PD and DLB had similar seeding activity. Consequently, receiver operating characteristic (ROC) curve analyses showed that Fl-αSyn and C-αSyn forms clearly differentiated PD or DLB from healthy controls using both TBS- and detergent-soluble fractions with 100% sensitivity and specificity (*p* = 0.02 for αSyn 1-140 and *p* = 0.007 for αSyn 1-130 and αSyn 1-115, Table 1a). However, none of the αSyn forms could differentiate PD from DLB using the temporal cortex. In the frontal cortex (Figure 3b), PD cases had greater seeding activity in the detergent-soluble fractions than in DLB cases. This was further illustrated by the ROC curve analysis of the frontal cortex data which showed that the detergent-soluble fraction, particularly with αSyn 1-130, could differentiate PD cases from DLB cases with 88% sensitivity and 100% specificity (*p* = 0.001, Table 1b, red font).

### 3.3. Detection of Proteinase K (PK)-Resistant Fragments of C-Terminal Truncated αSyn in the RT-QuIC End Products

**Figure 4:**
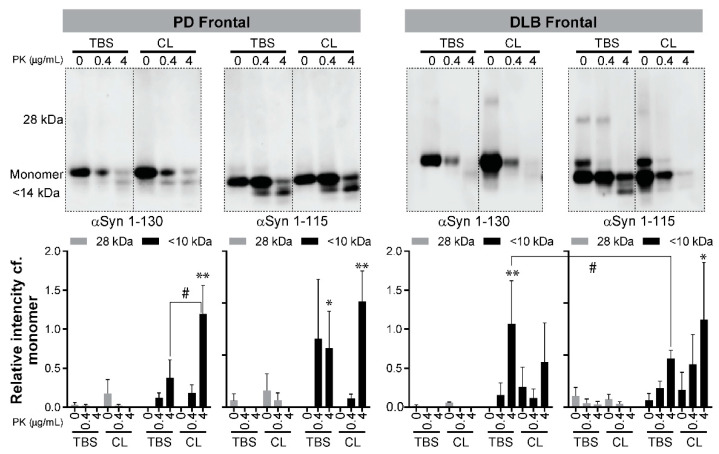
PK digestion of RT-QuIC end products from the frontal cortex of PD and DLB cases. The RT-QuIC end products (as described in Figure 2 and Figure 3) were collected and incubated at 37 °C with increasing concentrations of PK (0, 0.4, 4 µg/mL). The figure shows two panels where in each panel a representative immunoblot is shown for RT-QuIC PK digestion reactions, and a bar graph summarizing immunoblot quantification (mean + SD) from three cases for each condition. Immunoblots were quantified using ImageJ to calculate the intensity of aggregates (28 kDa) and PK-digested products (< 14 kDa) relative to the intensity of the monomeric αSyn in each lane. Statistical analysis was performed with GraphPad Prism using two-way ANOVA with Tukey’s post-test. Asterisks denote significant differences within a condition (* *p* < 0.05, ** *p* < 0.01) and hashtags denote differences across groups (# *p* < 0.05). Higher exposures of the blots are shown in Supplementary Figure S3 to show the high-molecular weight bands corresponding to αSyn aggregation.

**Figure 5:**
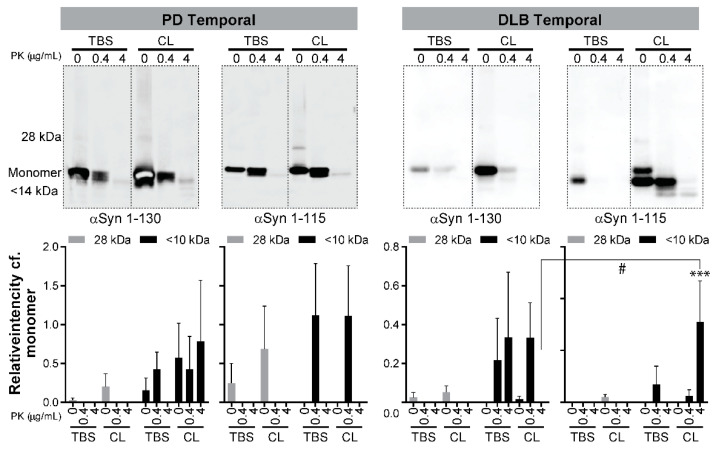
PK digestion of RT-QuIC end products from the temporal cortex of PD and DLB cases. The RT-QuIC end products (as described in Figure 2 and Figure 3) were collected and incubated at 37 °C with increasing concentrations of PK (0, 0.4, 4 μg/mL). The figure shows two panels where in each panel a representative immunoblot is shown for RT-QuIC PK digestion reactions, and a bar graph summarizing immunoblot quantification from three cases for each condition. Immunoblots were quantified using ImageJ to calculate the intensity of aggregates (28 kDa) and PK-digested products (<14 kDa) relative to the intensity of the monomeric αSyn in each lane. Statistical analysis was performed with GraphPad Prism using two-way ANOVA with Tukey’s post-test. Asterisks denote significant differences within a condition (*** *p* < 0.001) and hashtags denote differences across groups (# *p* < 0.05). Higher exposures of the blots are shown in Supplementary Figure S3 to show the high-molecular weight bands corresponding to αSyn aggregation.

Our results (Figure 3 and Table 1) concluded that C-αSyn130 is an attractive substrate for RT-QuIC assays. It differentiated DLB and PD from HC with 100% specificity and sensitivity using the TBS-soluble temporal cortex, and distinguished PD from DLB with 88% sensitivity and 100% specificity using the detergent-soluble frontal cortex. Thus, we next investigated if the end products from RT-QuIC assays using the different conditions had differences in resistance to proteinase K (PK) digestion. The conditions for PK digestions were deduced from PK digestion assays using recombinant monomeric αSyn as a negative control and in vitro aggregated αSyn as a positive control (see Supplementary Figure S2). Representative blots of PK digestion of end products from RT-QuIC assays with PD and DLB are shown for frontal cortex samples (Figure 4), and for temporal cortex samples (Figure 5). Using either of the C-αSyn forms, dimers (28kDa) were detected by immunoblotting before PK digestion and were not significantly different across PD and DLB brain homogenates (n = 3, Figure 4 and Figure 5, *p* > 0.05 two-way ANOVA).

For C- αSyn115, PK^res^ fragments were detected in detergent-soluble fractions from PD and DLB frontal cortex, but only in the aqueous-soluble fraction from PD frontal cortex (Figure 4, *p* < 0.05 vs. no PK digestion). For the temporal cortex, PK^res^ fragments from C-αSyn115 were detected only in the detergent-soluble fraction from DLB cases (Figure 5). There were no significant differences between PD and DLB in PK^res^ fragments in either cortex with C-αSyn115.

When comparing PD to DLB frontal cortex with C-αSyn130, PK^res^ fragments (< 14 kDa) in the TBS-soluble fractions were significantly higher in DLB versus PD (*p* < 0.05, Figure 4). On the other hand, the detergent-soluble fraction from PD frontal cortex with C-αSyn130 had higher, but not significant (*p* > 0.05), detection of PK^res^ bands compared to DLB cases. Moreover, with C-αSyn130, PK^res^ fragments were detected more significantly in the detergent-soluble fractions compared to the aqueous-soluble fractions in PD frontal cortex (Figure 4). These findings may support our finding (Table 1b) that C-αSyn130 with the detergent-soluble frontal cortex fractions was able to distinguish PD from DLB in RT-QuIC assays.

### 3.4. Morphological Characterization of RT-QuIC End Products by TEM

**Figure 6:**
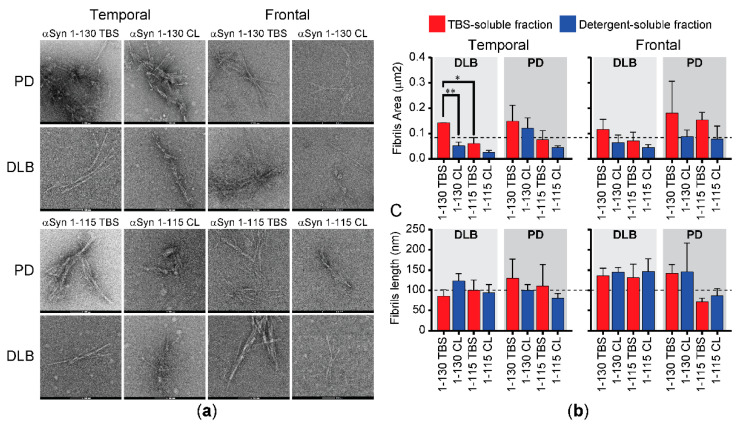
Morphological characterization of C-terminally truncated RT-QuIC end products by TEM. (**a**) Representative TEM images of RT-QuIC end products with the samples derived from PD and DLB temporal and frontal cortices. (**b**) Area of aggregated fibrils and (**c**) length of fibrils in each aggregate structure were calculated from the TEM images of the RT-QuIC end products using ImageJ; 5 to 10 fibrils per aggregate structure were measured to calculate the average fibril length per aggregate. Bar graphs show the average +SEM (n = 3 biological replicates (cases) per condition, except for PD TBS Frontal 1-115 where n = 2 cases). One-way ANOVA with Tukey’s multiple comparison testing (GraphPad Prism) was used for statistical comparisons; * *p* < 0.05, ** *p* < 0.01. The dotted lines in panels B and C show the average fibril area and fibril length from the temporal cortex samples, respectively.

The morphology of the RT-QuIC end products with truncated αSyn forms was analyzed by TEM. Representative images from single cases of PD and DLB are shown in Figure 6a. To quantify any observed morphological differences, we compared the average fibril length and the area of the entire aggregate (Figure 6b,c). TEM analysis did not show major differences between fibrils seeded with DLB or PD extracts. Overall, fibrils seeded with the detergent-soluble fractions appeared smaller in size than those seeded with the aqueous-soluble fraction, although the observed differences did not reach statistical significance, except for DLB temporal samples with αSyn 1-130 (*p* < 0.01, Figure 6b). The fibrils in the frontal cortex appeared longer than the temporal cortex, particularly with αSyn 1-130, but the differences were not statistically significant (Figure 6c).

## 4. Discussion

There is currently increasing interest in the potential role of distinct species of αSyn contributing to the diverse clinical features and clinical phenotypes of synucleinopathies, but relatively little is known about whether such species differ in terms of their expression and abundance across different brain regions and between distinct forms of synucleinopathies. Therefore, the present study sought to investigate whether different brain regions in PD and DLB could be distinguished in terms of their seeding capacity. We tested whether employing C-terminal truncated αSyn, which has been demonstrated to be more aggregation prone than full-length αSyn, may enable distinction of different brain regions from one another in synucleinopathies, or differences between synucleinopathies themselves, using the RT-QuIC assay. Here, we report that although temporal cortex tissue differentiated cases from controls in RT-QuIC, no differences in the seed-propensity of αSyn were observed across tissue fractions or between PD and DLB. In contrast, frontal cortex tissue did reveal differences in αSyn seeding, which was particularly efficient in distinguishing PD from DLB using detergent-soluble tissue fractions. We suggest these results indicate region- and phenotype-specific differences in αSyn-seeding propensity, which may relate to regionally diverse strains of αSyn, phenotype-associated αSyn pathology, or the maturity of aggregates between PD and DLB cortex.

Since the first report of aggregation assays, such as RT-QuIC for αSyn in both brain and CSF samples from patients affected by synucleinopathies [21], the RT-QuIC assay has been applied to a variety of biological samples, such as brain tissue samples, CSF, the olfactory mucosa [23], and the submandibular glands [24]. Despite these successful results, the ability of RT-QuIC in stratifying synucleinopathies is still controversial, with recent studies reporting conflicting results [10,12]. As standardization and harmonization of the αSyn RT-QuIC protocol between laboratories is lacking, it is possible that these different results are due to different experimental settings. For example, the use of different reaction buffers with different pH, the use of glass/silica beads, different shaking and rest times, and the purification method of the recombinant αSyn substrate, which can all influence the seeding kinetics and the robustness of the αSyn RT-QuIC assay [12,25]. Moreover, different brain regions may also harbor different strains of αSyn, reflecting the substantial diversity of neuronal populations, even across cortical regions. PD and DLB pathologies are both characterized by αSyn accumulation in cortical and subcortical regions, with DLB patients showing higher αSyn expression levels in the superior temporal cortex than PD and healthy control cases [26]. A small number of published studies comparing PD and DLB using RT-QuIC have found a higher seeding activity in DLB cases compared to PD cases when frontal cortex homogenates were used. This may be explained by the fact that deposition of αSyn aggregates in the frontal cortex is a prominent feature of DLB pathology, while in PD cases without dementia, the deposition is prominent in substantia nigra pars-compacta [9].

In the present study, we used the RT-QuIC assay to compare αSyn aggregation propensity between PD and DLB against healthy controls in two different brain regions, the frontal and temporal cortex, using aqueous (TBS)- and detergent-soluble fractions. The αSyn aggregation propensity in these brain fraction as seeds was evaluated using three different αSyn forms as substrates, Fl-αSy140 and C-terminally truncated (C-αSyn130 and C-αSyn115). To our knowledge, this is the first study to address αSyn aggregation propensity using different brain regions and substrates as a possible approach to differentiate between different αSyn strains.

We found that the RT-QuIC assay was unsuccessful using PBS (pH 7.4) as a reaction buffer, whereas PIPES (pH 6.9) was a more successful and reproducible conversion buffer. Remarkably, different fractions from distinct brain regions of PD and DLB cases showed different aggregation propensities, which was highlighted using a variety of αSyn forms as substrates. It should be noted that the differences observed between the aqueous- and detergent-soluble fractions in the frontal cortex are not related to the use of the detergent since there were no differences between these fractions in the temporal cortex. Moreover, previous studies have not evaluated RT-QuIC using the temporal cortex of synucleinopathies. In our study, RT-QuIC of temporal cortex lysates differentiated synucleinopathies from healthy controls, but the temporal cortex from PD and DLB had a similar aggregation propensity irrespective of the αSyn substrates or the nature of the fractions used. Consequently, RT-QuIC with the temporal cortex could differentiate PD and DLB cases from healthy controls with 100% sensitivity and specificity regardless of the fraction or recombinant αSyn substrate used.

In contrast, the frontal cortex of both PD and DLB cases showed higher aggregation propensity in the aqueous- versus detergent-soluble fractions. This is in line with our previous study with those same samples using ELISA [20], which showed that the levels of αSyn oligomers in the aqueous-soluble fraction are elevated in the frontal cortex of PD and DLB cases (700–750 pg/mL) compared to the detergent-soluble αSyn oligomers (250–300 pg/mL). Our previous ELISA study did not report differences in oligomeric αSyn levels between PD and DLB cases from either the aqueous- or detergent-soluble frontal cortex fractions [20]. This was confirmed by our study here using RT-QuIC. However, it should be noted that another study using RT-QuIC reported a higher seeding activity in the frontal cortex of DLB cases compared to frontal cortex of PD cases [9]. This discrepancy may be explained by differences in the brain homogenate preparation. Candelise et al. [9] subjected the total frontal cortex homogenates to a complex, centrifugation-based purification method to isolate >100 kDa αSyn seeds from the samples, while our previous study [20] and this study employed direct extraction methods.

In the frontal cortex, we found that the aggregation propensity increased with increasing C-terminal truncation of αSyn substrates. Interestingly, while αSyn 1-140 differentiated PD and DLB from healthy controls with 100% sensitivity and specificity, the C-αSyn forms also demonstrated differences between PD and DLB. Remarkably, αSyn 1-130 as substrate with detergent-soluble fractions from the frontal cortex differentiated PD from DLB with 88% sensitivity and 100% specificity. Altogether, our data show that RT-QuIC using αSyn 1-130 substrate with the temporal cortex could differentiate PD and DLB from healthy controls, and it can differentiate PD from DLB using the detergent-soluble fraction from the frontal cortex. In contrast, αSyn 1-115 as substrate did not distinguish PD from DLB in both brain regions. However, αSyn 1-115 differentiated PD and DLB from healthy controls with 100% sensitivity and 100% specificity with the aqueous-soluble fractions, and with 93% sensitivity and 100% specificity with the detergent-soluble fractions from the temporal cortex.

One might speculate that the presence of αSyn strains with different cellular tropism might have the ability to transmit their conformational properties to the recombinant amyloid fibrils in RT-QuIC. In agreement with this observation, Shahnawaz et al. showed that the conformational properties of αSyn aggregates associated with PD and MSA can be transmitted by αSyn-PMCA [10]. In this study, end products from RT-QuIC reactions using the C-terminally truncated αSyn forms showed more obvious PK^res^ fragments in the frontal cortex than in the temporal cortex, suggesting the existence of αSyn strains with different biochemical properties, perhaps reflecting regional differences in cellular composition. TEM analysis in this study suggested that reaction end products seeded with the aqueous-soluble fractions were larger in size than those from reactions seeded with the detergent-soluble fractions, which agrees with the increased seeding response observed in RT-QuIC compared to full-length αSyn. Moreover, the fibrils detected by TEM of the RT-QuIC end products appeared longer in the frontal cortex than the temporal cortex. Taken together, these findings strongly suggest that αSyn aggregates show substantial heterogeneity even within individuals, with important implications for understanding the pathobiology of αSynucleinopathies, the development of therapeutics targeting αSyn, and personalized medicine.

Several studies have identified different C-terminal truncated forms of αSyn in αSynucleinopathies, and different aggregation properties of truncated forms have been studied in vitro [18]. While αSyn forms have been quantified in brains of PD patients [27], to the best our knowledge, few studies have compared the presence of C-terminal truncated αSyn forms in different αSynucleinopathies and in different brain regions. Interestingly, it has been reported that truncated αSyn is present in 70% of Lewy bodies and neurites in DLB versus 90% in PD [28]. Moreover, another study has shown different levels of C-terminal truncated αSyn forms in diffused Lewy body disease (DLBD) and Alzheimer’s disease with Lewy body pathology [11]. Our study did not directly investigate C-terminal truncated αSyn forms as seeds but measured different aggregation propensities by the RT-QuIC assay in different brain regions and extracts from PD and DLB. Those findings emphasize the need to determine if those differences are related to the amounts and types of C-terminal truncated αSyn forms in different αSynucleinopathies.

One limitation of using the RT-QuIC and the PMCA assays to measure αSyn aggregation propensity in biological samples is the inability to characterize the actual strains and seeds in samples. In our study, using different substrates, we reported heterogeneity in the αSyn aggregation propensity between different brain regions and extracts from PD and DLB patients. However, it remains unclear whether those differences are driven by specific endogenous differences in samples, which include different nature (forms) and amounts of the αSyn strains and seeds. The actual strains and seeds that cause the propagation of αSyn aggregation is of a great interest in the field of αSynucleinopathies. Quantification of different forms of αSyn—full length versus truncated, phosphorylated versus non-phosphorylated, and monomeric versus oligomeric versus fibrils—may allow testing correlations between different αSyn forms in biological samples and in vitro aggregation propensity. Importantly, specific depletion of different αSyn forms in the sample is required to determine which form(s) cause the aggregation in the RT-QuIC assays but such studies cannot be conducted at present due to the lack of tools such as antibodies specific to the different αSyn forms. Nonetheless, it should be noted that one advantage of the RT-QuIC technique is its strain/seed-agnostic nature which allows the comparison of seeding propensity across biological samples in a semi-quantitative manner as deployed in our study.

In conclusion, our study demonstrated the value of using C-terminally truncated αSyn 1-130 and αSyn 1-115 as substrates for RT-QuIC assays to differentiate PD and DLB from healthy controls and differentiating PD from DLB. Biochemical characterization of the end products from RT-QuIC, particularly with the αSyn 1-130 protein, suggests differences in PK resistance and αSyn aggregate size and fibril length depending on the brain regions and fractions used. Further studies are warranted to investigate αSyn 1-130, and C-terminal truncated αSyn other than 1-115, to differentiate synucleinopathies by their seeding propensity using RT-QuIC. 

Different strategies have been used to improve the sensitivity and the specificity of the αSyn RT-QuIC assay for the clinical diagnosis of synucleinopathies using CSF as seed. However, one of the major drawbacks remains the duration of the reaction. A single RT-QuIC experiment using full-length αSyn takes around five days to reach completion, which is not ideal in a clinical setting. In our study, we have observed a trend towards faster kinetics of aggregation with increasing truncation of α-Syn using brain homogenates fractions as seed. The use of truncated αSyn forms as substrates should be exploited to improve the diagnostic utility of the RT-QuIC assay.

## Figures and Tables

**Table 1:** ROC curve analysis of RT-QuIC data with PD and DLB cases with different αSyn forms.

**a**	**TBS-Soluble Fraction**				**Detergent-Soluble Fraction**			
**Temporal**	**Sens.**	**Spec.**	*p* *	**Cutoff**	**Sens.**	**Spec.**	p *	**Cutoff**
				**(RT-QuIC AUC)**				**(RT-QuIC AUC)**
	**αSyn 1-140**							
Cases vs. HC	100%	100%	0.006	>5,686,027	100%	100%	0.007	>4,010,801
PD vs. HC	100%	100%	0.02	>5,686,027	100%	100%	0.02	>5,343,731
DLB vs. HC	100%	100%	0.02	>5,889,846	100%	100%	0.02	>4,010,801
PD vs. DLB	75%	50%	0.563	<14,474,992	100%	75%	0.043	>10,077,548
	**αSyn 1-130**							
Cases vs. HC	100%	100%	0.003	>1,957,147	94%	100%	0.003	>1,367,850
PD vs. HC	100%	100%	0.007	>2,775,490	100%	100%	0.007	>700,526
DLB vs. HC	100%	100%	0.007	>1,957,147	88%	100%	0.007	>1,404,507
PD vs. DLB	75%	50%	0.834	<16,198,128	88%	50%	0.401	>3,158,315
	**αSyn 1-115**							
Cases vs. HC	100%	100%	0.003	>5,198,217	94%	100%	0.003	>6,530,860
PD vs. HC	100%	100%	0.007	>5,198,217	100%	100%	0.007	>4,395,742
DLB vs. HC	100%	100%	0.007	>6,335,727	100%	100%	0.007	>2,164,070
PD vs. DLB	63%	75%	0.142	<14,687,906	63%	63%	0.207	<13,688,774
**b**	**TBS-soluble fraction**				**Detergent-soluble fraction**			
**Frontal**	**Sens.**	**Spec.**	*p* *	**Cutoff**	**Sens.**	**Spec.**	*p* *	**Cutoff**
				**(RT-QuIC AUC)**				**(RT-QuIC AUC)**
	**αSyn 1-140**							
Cases vs. HC	100%	100%	0.006	<35,870	100%	100%	0.006	>64.66
PD vs. HC	100%	100%	0.02	>515,271	100%	100%	0.02	>72.59
DLB vs. HC	100%	100%	0.02	>35,870	100%	100%	0.006	>64.66
PD vs. DLB	75%	100%	0.25	<893,180	100%	75%	0.15	<200,163
	**αSyn 1-130**							
Cases vs. HC	100%	100%	0.001	>5,142,617	100%	50%	0.024	0.7891
PD vs. HC	100%	100%	0.001	>5,142,617	88%	88%	0.003	0.9375
DLB vs. HC	88%	88%	0.005	>4,666,272	88%	50%	0.345	>318,073
PD vs. DLB	75%	88%	0.093	>10,642,878	88%	100%	0.001	<957,841
	**αSyn 1-115**							
Cases vs. HC	88%	75%	0.006	>11,421,461	87%	88%	0.001	>673,667
PD vs. HC	75%	88%	0.021	>13,701,264	88%	88%	0.002	>1,205,104
DLB vs. HC	88%	75%	0.016	>11,421,461	88%	88%	0.009	>673,667
PD vs. DLB	88%	50%	0.528	<15,596,073	75%	75%	0.248	<4,442,097

* Statistical differences for the AUC compared to 0.5 using C-statistic were performed using GraphPad Prism.

## Data Availability

All data relevant to this work are presented in full.

## Supplementary Materials

The following are available online at https://www.mdpi.com/article/10.3390/biom11060820/s1, Supplementary Table: Clinical characteristics of the PD, DLB, and healthy control cases used in the study; Figure S1: Unseeded RT-QuIC reactions; Figure S2: PK digestion of recombinant αSyn forms in the pure form (monomeric) and after in vitro aggregation of the recombinant αSyn (aggregates); Figure S3: High exposure of blots in Figure 4 and Figure 5 for the PK digestion of RT-QuIC end products from the frontal (top) and temporal (bottom) cortices of PD and DLB cases.

## References

[1] McCann H., Stevens C.H., Cartwright H., Halliday G.M. (2014). alpha-Synucleinopathy phenotypes. Parkinsonism Relat. Disord..

[2] Spillantini M.G., Crowther R.A., Jakes R., Hasegawa M., Goedert M. (1998). alpha-Synuclein in filamentous inclusions of Lewy bodies from Parkinson’s disease and dementia with lewy bodies. Proc. Natl. Acad. Sci. USA.

[3] Halliday G.M., Song Y.J.C., Harding A.J. (2011). Striatal beta-amyloid in dementia with Lewy bodies but not Parkinson’s disease. J. Neural Transm..

[4] Peelaerts W., Bousset L., Van der Perren A., Moskalyuk A., Pulizzi R., Giugliano M., Van den Haute C., Melki R., Baekelandt V. (2015). alpha-Synuclein strains cause distinct synucleinopathies after local and systemic administration. Nature.

[5] Lee S.-J., Masliah E. (2015). Neurodegeneration: Aggregates feel the strain. Nature.

[6] Campbell B.C.V., McLean C.A., Culvenor J.G., Gai W.P., Blumbergs P.C., Jäkälä P., Beyreuther K., Masters C.L., Li Q.-X. (2001). The solubility of alpha-synuclein in multiple system atrophy differs from that of dementia with Lewy bodies and Parkinson’s disease. J. Neurochem..

[7] Van der Perren A., Gelders G., Fenyi A., Bousset L., Brito F., Peelaerts W., Van den Haute C., Gentleman S., Melki R., Baekelandt V. (2020). The structural differences between patient-derived alpha-synuclein strains dictate characteristics of Parkinson’s disease, multiple system atrophy and dementia with Lewy bodies. Acta Neuropathol..

[8] Sorrentino Z.A., Goodwin M.S., Riffe C.J., Dhillon J.-K.S., Xia Y., Gorion K.-M., Vijayaraghavan N., McFarland K.N., Golbe L.I., Yachnis A.T. (2019). Unique alpha-synuclein pathology within the amygdala in Lewy body dementia: Implications for disease initiation and progression. Acta Neuropathol. Commun..

[9] Candelise N., Schmitz M., Llorens F., Villar-Piqué A., Cramm M., Thom T., da Silva Correia S.M., da Cunha J.E.G., Möbius W., Outeiro T.F. (2019). Seeding variability of different alpha synuclein strains in synucleinopathies. Ann. Neurol..

[10] Shahnawaz M., Mukherjee A., Pritzkow S., Mendez N., Rabadia P., Liu X., Hu B., Schmeichel A., Singer W., Wu G. (2020). Discriminating alpha-synuclein strains in Parkinson’s disease and multiple system atrophy. Nature.

[11] Lewis K.A., Su Y., Jou O., Ritchie C., Foong C., Hynan L.S., White C.L., Thomas P.J., Hatanpaa K.J. (2010). Abnormal neurites containing C-terminally truncated alpha-synuclein are present in Alzheimer’s disease without conventional Lewy body pathology. Am. J. Pathol..

[12] Rossi M., Candelise N., Baiardi S., Capellari S., Giannini G., Orru C.D., Antelmi E., Mammana A., Hughson A.G., Calandra-Buonaura G. (2020). Ultrasensitive RT-QuIC assay with high sensitivity and specificity for Lewy body-associated synucleinopathies. Acta Neuropathol..

[13] Liu C.W., Giasson B.I., Lewis K.A., Lee V.M., Demartino G.N., Thomas P.J. (2005). A precipitating role for truncated alpha-synuclein and the proteasome in alpha-synuclein aggregation: Implications for pathogenesis of Parkinson disease. J. Biol. Chem..

[14] Murray I.V., Giasson B.I., Quinn S.M., Koppaka V., Axelsen P.H., Ischiropoulos H., Trojanowski J.Q., Lee V.M. (2003). Role of alpha-synuclein carboxy-terminus on fibril formation in vitro. Biochemistry.

[15] Periquet M., Fulga T., Myllykangas L., Schlossmacher M.G., Feany B.M. (2007). Aggregated alpha-synuclein mediates dopaminergic neurotoxicity in vivo. J. Neurosci..

[16] Michell A.W., Tofaris G.K., Gossage H., Tyers P., Spillantini M.G., Barker R.A. (2007). The effect of truncated human alpha-synuclein (1-120) on dopaminergic cells in a transgenic mouse model of Parkinson’s disease. Cell Transpl..

[17] Baba M., Nakajo S., Tu P.H., Tomita T., Nakaya K., Lee V.M., Trojanowski J.Q., Iwatsubo T. (1998). Aggregation of alpha-synuclein in Lewy bodies of sporadic Parkinson’s disease and dementia with Lewy bodies. Am. J. Pathol..

[18] Sorrentino Z.A., Vijayaraghavan N., Gorion K.M., Riffe C.J., Strang K.H., Caldwell J., Giasson B.I. (2018). Physiological C-terminal truncation of alpha-synuclein potentiates the prion-like formation of pathological inclusions. J. Biol. Chem..

[19] Chakroun T., Evsyukov V., Nykanen N.P., Hollerhage M., Schmidt A., Kamp F., Ruf V.C., Wurst W., Rosler T.W., Hoglinger G.U. (2020). Alpha-synuclein fragments trigger distinct aggregation pathways. Cell Death Dis..

[20] Vaikath N.N., Erskine D., Morris C.M., Majbour N.K., Vekrellis K., Li J.Y., El-Agnaf O.M.A. (2019). Heterogeneity in alpha-synuclein subtypes and their expression in cortical brain tissue lysates from Lewy body diseases and Alzheimer’s disease. Neuropathol. Appl. Neurobiol..

[21] Fairfoul G., McGuire L.I., Pal S., Ironside J.W., Neumann J., Christie S., Joachim C., Esiri M., Evetts S.G., Rolinski M. (2016). Alpha-synuclein RT-QuIC in the CSF of patients with alpha-synucleinopathies. Ann. Clin. Transl. Neurol..

[22] Koss D.J., Dubini M., Buchanan H., Hull C., Platt B. (2018). Distinctive temporal profiles of detergent-soluble and -insoluble tau and Abeta species in human Alzheimer’s disease. Brain Res..

[23] De Luca C.M.G., Elia A.E., Portaleone S.M., Cazzaniga F.A., Rossi M., Bistaffa E., De Cecco E., Narkiewicz J., Salzano G., Carletta O. (2019). Efficient RT-QuIC seeding activity for alpha-synuclein in olfactory mucosa samples of patients with Parkinson’s disease and multiple system atrophy. Transl. Neurodegener..

[24] Manne S., Kondru N., Jin H., Anantharam V., Huang X., Kanthasamy A., Kanthasamy A.G. (2020). alpha-Synuclein real-time quaking-induced conversion in the submandibular glands of Parkinson’s disease patients. Mov. Disord..

[25] Candelise N., Schmitz M., Thune K., Cramm M., Rabano A., Zafar S., Stoops E., Vanderstichele H., Villar-Pique A., Llorens F. (2020). Effect of the micro-environment on alpha-synuclein conversion and implication in seeded conversion assays. Transl. Neurodegener..

[26] Koob A.O., Shaked G.M., Bender A., Bisquertt A., Rockenstein E., Masliah E. (2014). Neurogranin binds alpha-synuclein in the human superior temporal cortex and interaction is decreased in Parkinson’s disease. Brain. Res..

[27] Kellie J.F., Higgs R.E., Ryder J.W., Major A., Beach T.G., Adler C.H., Merchant K., Knierman M.D. (2014). Quantitative measurement of intact alpha-synuclein proteoforms from post-mortem control and Parkinson’s disease brain tissue by intact protein mass spectrometry. Sci. Rep..

[28] Dufty B.M., Warner L.R., Hou S.T., Jiang S.X., Gomez-Isla T., Leenhouts K.M., Oxford J.T., Feany M.B., Masliah E., Rohn T.T. (2007). Calpain-cleavage of alpha-synuclein: Connecting proteolytic processing to disease-linked aggregation. Am. J. Pathol..

