# Supplementary material for: RT-QuIC Using C-Terminally Truncated α-Synuclein Forms Detects Differences in Seeding Propensity of Different Brain Regions from Synucleinopathies
Source: Biomolecules. 2021 May 31;11(6):820. doi: 10.3390/biom11060820 (PMC8226794; doi:10.3390/biom11060820)
Supplement: Supplementary file 1 [file biomolecules-11-00820-s001.zip › biomolecules-1201760-supplementary.pdf]

Supplementary Table. Clinical characteristics of the PD, DLB and healthy control cases used in the study

Case ID	Diagnosis	Age	Sex	PMI (h)	Tissue pH	Braak NFT	Braak LB
C1	Control	89	F	24	N.D.	2	1
C2	Control	75	M	20	N.D.	0	0
C3	Control	69	F	16	N.D.	1	0
C4	Control	72	F	28	6.86	1	0
C5	Control	65	M	14	N.D.	1	0
C6	Control	72	M	17	6.13	1	0
C7	Control	87	M	8	5.69	2	0
C8	Control	87	F	14	6.63	4	N.D.
P1	PD	74	F	16	6.08	N.D.	N.D.
P2	PD	84	M	38	N.D.	N.D.	N.D.
P3	PD	68	M	18	N.D.	0	N.D.
P4	PD	67	F	38	N.D.	0	N.D.
P5	PD	78	M	24	N.D.	0	5
P6	PD	69	M	35	N.D.	0	4
P7	PD	82	M	7	5.91	2	5
P8	PD	64	M	33	N.D.	0	5
D1	DLB	88	M	4	N.D.	2	6
D2	DLB	77	M	8	N.D.	2	6
D3	DLB	79	M	26	6.66	2	5
D4	DLB	77	F	23	6.24	4	6
D5	DLB	72	M	18	6.29	3	6
D6	DLB	85	F	18	N.D.	4	6
D7	DLB	79	F	29	5.75	3	6
D8	DLB	75	M	18	6.45	2	5

Abbreviations. **PMI (h)**: post mortem interval in hours; **Braak NFT**: Braak neurofibrillary tangle staging; **Braak LB**: Braak Lewy bodies staging

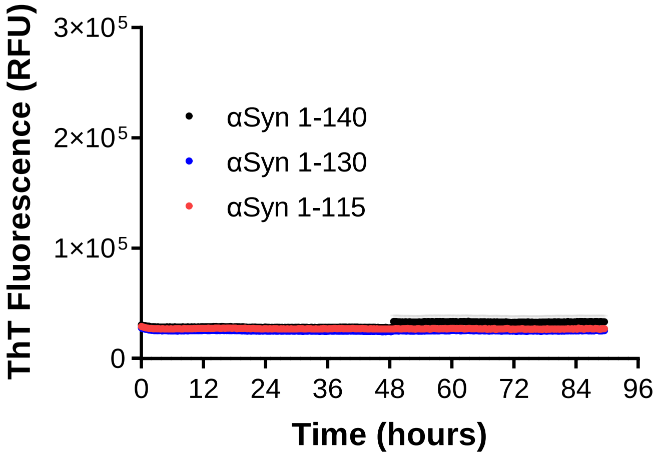

Figure S1: Unseeded RT-QuIC reactions. RT-QuIC assay using recombinant full-length αSyn (1-140) or each of the two C-terminally truncated forms (1-130 and 1-115) alone; i.e. unseeded reactions. Data shown is the average ThT fluorescence over time (n = 3, ± SEM).

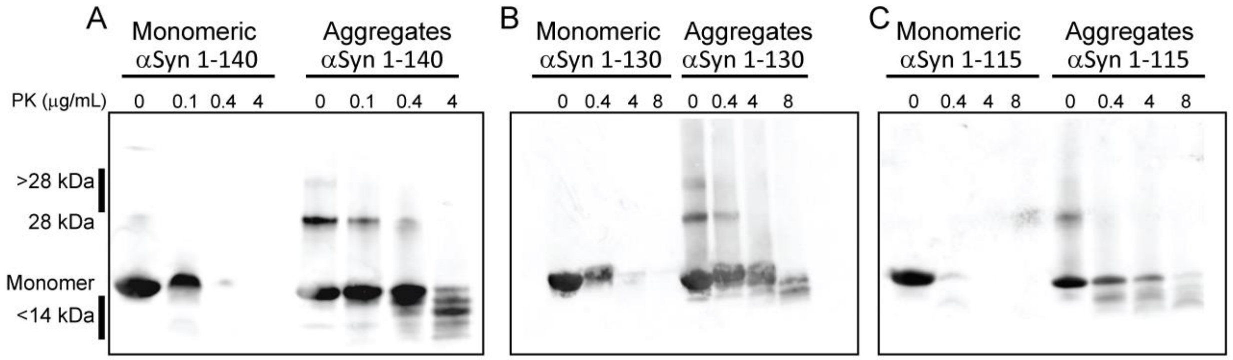

Figure S2. PK digestion of recombinant αSyn forms in the pure form (monomeric) and after in vitro aggregation of the recombinant αSyn (aggregates). Full length αSyn 1-140 and C-terminally truncated αSyn (1-130 and 1-115) were aggregated *in vitro*. Aggregation was carried out using 1 mg/mL solution of recombinant proteins in PBS by incubation at 37ºC for 7 days with constant shaking at 800 rpm. Aggregates were collected by centrifugation at 14'000 rpm for 15 min at 4ºC, then pellets were washed twice with 500 µL PBS and centrifugation 14'000 rpm for 15 min at 4ºC. The final pellets were resuspended in 300 µL of PBS. The concentration of the samples was determined by BCA assay and stored at -80ºC. Monomeric αSyn and aggregates prepared from αSyn 1-140 (**A**), αSyn 1-130 (**B**) and (**C**) αSyn 1-115 were incubated at 37ºC with increasing concentrations of PK for 30 minutes before immunoblotting with Syn-1 antibody.

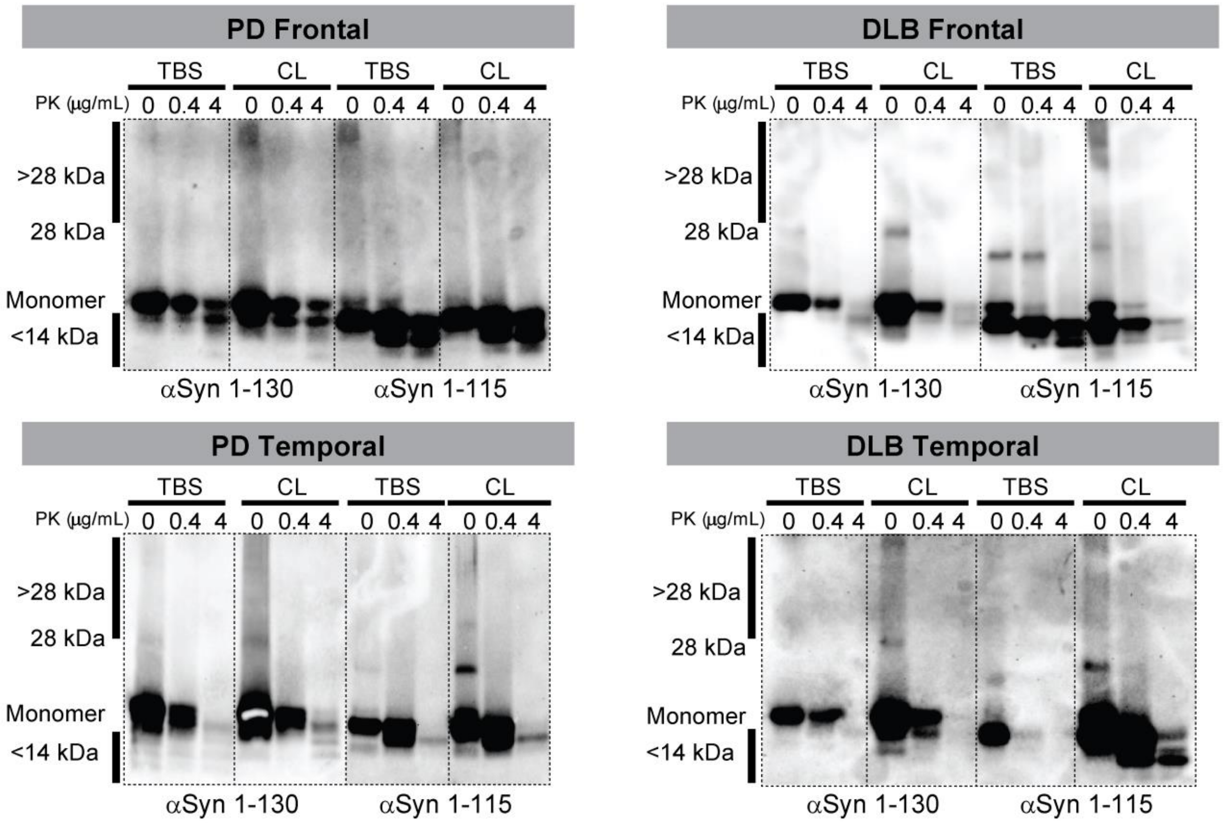

Figure S3. High exposure of blots in Figure 4 and Figure 5 for the PK digestion of RT-QuIC end products from the frontal (top) and temporal (bottom) cortices of PD and DLB cases.
